# Depressive Symptoms among Chinese Informal Employees in the Digital Era

**DOI:** 10.3390/ijerph18105211

**Published:** 2021-05-14

**Authors:** Yang Cai, Weiwei Kong, Yongsheng Lian, Xiangxin Jin

**Affiliations:** 1 School of Public Finance and Administration, Harbin University of Commerce, Harbin 150001, China; caiyang29@163.com (Y.C.); lianyongsheng664@163.com (Y.L.); kinxim@163.com (X.J.); 2 School of Accounting, Harbin University of Commerce, Harbin 150001, China

**Keywords:** CFPS, China, depressive symptoms, digital economy, informal employment, Internet use, quantitative analysis

## Abstract

The mental health status of informal employees is rarely studied in China. Nowadays, new economic forms such as gig economy and platform economy are emerging with the rapid development of information and communication technology, which has brought great changes to the labor market, especially to the informal employment field. Thus, it is of great significance to investigate the depressive symptoms among informal employees in the digital era. Based on the cross-sectional data of CFPS (China Family Panel Studies, 2018), this study takes a quantitative analysis framework to explore and analyze the association between informal employment and depressive symptoms in the Chinese labor market. After screening, a data set of 8893 employees (60.5% male and 39.5% female) was established. Several statistical methods, including the Mann–Whitney test and probit regression model, were used in the sample data analysis. The results show that the prevalence of depressive symptoms among informal employees is significantly higher than that among formal employees. Depressive symptoms are highly related to informal work and other factors, such as education, physical health, household income, etc. The impact of Internet use on informal employees’ depressive symptoms is not significant. The mental health inequality between formal and informal employees still exists in the digital era, and corresponding labor market regulations and social policies should be perfected to address this issue.

## 1. Introduction

The term informal employment mainly refers to the workers in informal sectors (a subset of unincorporated enterprises not constituted as separate legal entities independently of their owners) and those in the formal economy but in informal work arrangements [1]. With the global integration and shifts in economic policies, informal employment has become the mainstream of today’s labor market all over the world. The ILO (International Labour Organization) reports that about two billion workers are in informal employment, representing 61.2% of the world’s employed population in 2018 [2]. In China, the transformation trend also exists. Casual employment, non-standard contractual arrangements, and informal jobs are replacing full-time employment in formal sectors. Although there are no official statistics on the size of informal employment, several empirical evidences show that over half of the workforce in the urban labor market engaged in informal jobs in the 2010s [3,4].

Informal employment is usually related to low income and social insurance coverage, poor working conditions, and limited safety protection, which will affect the physical and mental health of employees in many aspects. Except for few individual cases, the vulnerability of informal employment has been verified in a number of literatures based on data from different countries [5,6,7,8,9,10,11]. A systematic review covering 12 studies in four WHO (World Health Organization) regions (Africa, the Americas, the Eastern Mediterranean, and the Western Pacific) shows that informal economy workers may be less likely than formal economy workers to use any health services and more likely to have depression [12]. Traditionally, the majority of the informal labor force in China is the surplus labor force migrating from rural areas to urban areas. Due to the lack of education and working experiences, the migrated workers often engage in temporary manual working with relatively low income, and they are often regarded as vulnerable groups [13,14].

In recent years, the rapid development of ICT (Information and Communication Technology) and popularity of mobile Internet has a significant impact on the labor market. The emergence of new economic forms, such as gig economy, sharing economy, and digital platform economy, have brought great changes to many traditional industries [15,16,17,18]. Meanwhile, a number of related jobs are emerging in the labor market, such as online car-hailing driver, delivery rider, take-away rider, and O2O (Online-to-Offline) service personnel, which usually belong to the informal employment field [19,20]. By December 2018, the number of Internet users in China reached 829 million, 98.6% of which were mobile Internet users, and there were 191 million employees working in the digital economy field, accounting for 24.6% of the total employment population [21,22]. With the progress of labor productivity, the Chinese economic structure is shifting from the secondary industry to the tertiary industry, which makes more workers shift from manufacturing factories to other sectors. The concepts of people’s attitudes towards job hunting is also changing. Informal employment and entrepreneurship have become the voluntary choices of many college students. Researchers hold different views on the influences of informal employment in new economic forms [15,16,17,23,24,25,26,27]. Supporters claim that employees may have high levels of flexibility and autonomy and low intensity of physical labor, which will increase their income and improve their working conditions and occupational health, while opponents state that there is no essential distinction in the digital forms from traditional informal employment.

As the main objective of the Chinese labor market regulation changes from providing more job opportunities to improving employment quality, the government pays more and more attention to improving the occupational health status and well-being of employees [28,29]. At present, the working condition, income inequality, labor relation, and regulation of informal employment has been widely concerned by researchers in China, and some studies involve the occupational health of informal employees [3,30,31,32,33]. However, only few studies involve the mental health status of Chinese informal employees before [34,35]. Ding takes subjective well-being as the mental health index and finds that residents engaged in informal employment have significantly lower subjective well-being; there is serious gender discrimination in the Chinese informal employment market, which significantly reduces women’s subjective well-being; the well-being of middle-aged residents is relatively low [34]. Wu et al. find that compared to women working in the public sector or not in the labor market, women working in the private sector spend more hours per week in paid work, and their subjective well-being is considerably low [35].

There are many manifestations of mental health, including subjective well-being, anxiety, stress, depression, and so on. They all reflect people’s psychological state from different aspects. Up to now, depressive symptoms among informal employees are rarely studied in China; there is especially a lack of empirical research on the impact of the digital economy on employees’ mental health.

Based on the micro data from a CFPS (China Family Panel Studies) survey in 2018, this study intends to use several statistical methods to empirically and quantitatively explore the mental health status of informal employees in the digital economy era. This study focuses on the two aspects: (1) the difference in depressive symptoms between informal and formal employees and its influencing mechanism; and (2) the impact of Internet use on depressive symptoms of informal employees.

## 2. Materials and Methods

### 2.1. Data Sources and Sampling

The research data are obtained from CFPS, which is a nationwide, comprehensive, longitudinal social survey conducted by the Institute of Social Science Survey, Peking University [36,37]. The CFPS 2018 survey started in June 2018 and ended in May 2019. A total of 15,000 families were interviewed, and about 44,000 questionnaires were collected. The questionnaires covered 31 provinces in China, and the multi-stage sample strategy in CFPS is well-designed as stated in [37]; thus, CFPS can be considered nearly nationally representative.

Considering the particularity in terms of concepts, classifications, survey content, and statistical measures of agricultural work, agricultural workers are not involved in this study. Furthermore, since CFPS records the income of self-employment by family, it is difficult to separate personal income from the household income. Thus, this study only involves non-agricultural employees, and the self-employment observations are not included. From 37,354 self-administered questionnaires, an initial data set was firstly established containing all the employees that took the depression test completely and were aged between 18–65. Then, a tag variable was added to mark if one observation belonged to informal employment or not. The rules for distinguishing between formal and informal employment are shown as follows:

(1) Employees who sign labor contracts and have social insurance (including endowment insurance and medical insurance) are considered to be formal employment.
(2) Considering the Chinese unique housing provident fund system, employees working in state-owned enterprises, government agencies, and public institutions are regarded as having formal employment as long as they have the housing provident fund, whether they sign labor contracts or not. In fact, many lifelong employees in the public sectors did not sign labor contracts in the past; only new employees and temporary workers signed labor contracts, oppositely.
(3) On the basis of the above screening rules, if an employee signs a labor dispatch or labor intermediary contract, which means the organization providing the labor contract is inconsistent with the organization he/she actually works for, this observation is regarded as informal and deleted from the formal data set.
(4) Abnormal observations are excluded manually if the key variable is missing or exceeds normal range.

After the above steps, a total set of 8893 observations was established, among which 2984 observations were identified as formal employment, and the remaining 5909 observations were identified as informal employment. Comparing with other statistics reported in [3,4,34], the proportion of informal employment was a bit higher (66.4%) because the screening rules of formal employment in this study are relatively strict.

### 2.2. Measures of Depression

The depressive symptoms of CFPS participants were evaluated by the Center for Epidemiologic Studies Depression scale (CES-D). CES-D is one of the most widely used self-evaluation depression scales, originally developed by Radloff in 1977 [38]. The standard CES-D scale contains 20 items. In order to shorten the answer time and improve the response rate, CFPS began to use the simplified 8-item CES-D version from 2016. Previous studies show that the 8-item CES-D scale is a valid and reliable screening measure of depressive symptoms [39,40,41,42].

In the 8-item CES-D scale, six items measure negative feelings (“I felt depressed”, “I felt that everything I did was an effort”, “My sleep was restless”, “I felt lonely”, “I felt sad”, and “I could not get going”) and two items measure positive feelings (“I was happy” and “I enjoyed life”). The participant is asked to assess the frequency of certain moods during the past week and choose one response from the four proposed answers: “rarely or none of the time (<1 day)”, “some or a little of the time (1–2 days)”, “occasionally or a moderate amount of time (3–4 days)”, and “most or all of the time (5–7 days)”. The responses to the negative items are assigned to an index value of 0, 1, 2, and 3, while the responses to positive items are assigned as 3, 2, 1, and 0. In total, an overall CES-D score ranging from 0 to 24 is accumulated, with higher score indicating greater depressive symptoms.

The Cronbach’s alpha test was used to measure the internal consistency of the 8-item CES-D scale in this study. The Cronbach’s alpha value based on standardized items was 0.7616, which is lower than the original 20-item CES-D scale (about 0.85, reported in [38]). The possible explanation may be the less items, the regional and demographic differences, etc. Since the alpha value is above 0.7, the results of the scale are still considered to be trustworthy.

The CES-D classification of depressive symptoms might vary across countries and population groups. When using standard 20-item CES-D scale, Radloff suggested a score of 16 or the 80th percentile point as the cutoff point to indicate highly suspect depressive symptoms [38]. In this study, the 80th percentile point (score of 8) of the 8-item CES-D total score is used as the cut-off point to assess whether the participant is in high-risk depressive symptoms or not. In the follow-up statistical analysis, both the CES-D score distribution and prevalence of depressive symptoms were used to compare the mental health difference between subsamples.

### 2.3. Variables Selection in the Regression Model

Previous studies show that depression might be related to personal characteristics, living environment, working conditions, marital status, wealth, social relationship, and genetic factors, etc. [43,44,45,46]. In fact, the factors related to individual depressive symptoms may vary from each other. In this study, several regression models were used to analyze the correlation between depression and possible causing factors. Table 1 lists the selected variables in the regression model and gives the description and value range of the variables. In particular, this study used working with Internet as the representation of participating in digital economy. In the regression model, *cesd* and *depress* are the dependent variables and *informal* and *internet* are the independent variables. Several control variables are selected in addition, including the participant’s gender, age, education, marital status, perceived health, personal and household annual income, perceived interpersonal relationship, and social class.

### 2.4. Statistical Analysis

The statistical analysis contains three steps. In step I, the descriptive statistics of the samples were computed and graphed. In step II, the difference in depressive symptoms between informal and formal employees were analyzed to explore whether there is a mental health inequality in Chinese labor market. Both the inter-group difference test and multivariable regression analysis methods were used. In step III, informal employees working with or without Internet were divided into two subsamples, and the difference in depressive symptoms between the two subsamples were analyzed to explore the impact of Internet-related work on the mental health status of informal employees.

In step II, since the CES-D score presented right-skewness distribution in all the data sets, the non-parametric statistical method of the Mann–Whitney test was performed to explore whether there was a statistically significant difference between the two subsamples (significance level set at *p* < 0.05). Then, the multivariable analysis was taken. An ordered probit model was used to explore the association between the dependent variable (*cesd*) and independent variable (*informal*) and other control variables. The reason why the ordered probit model was used instead of OLS (Ordinary Least Squares) is that *cesd* is not a continuous variable but an ordered variable. A binary probit model was also used to check the robustness of the regression results, in which the dependent variable was *depress*. In step III, the statistical analysis methods and procedures were similar with that in step II, except the independent variable in probit regression model was replaced by *internet*.

The regression model in step II is stated in Equation (1), and the regression model in step III is stated in Equation (2):

(1) $$y_{i}{}^{*}=\beta_{1}\times informal_{i}+\gamma_{1}\times X_{i}+\varepsilon_{i}$$

(2) $$y_{i}{}^{*}=\beta_{2}\times internet_{i}+\gamma_{2}\times X_{i}+\varepsilon_{i}$$

where $y_{i}{}^{*}$ is the estimation of dependent variables (*cesd* and *depress*); $informal_{i}$ is the independent variable in step II; $internet_{i}$ is the independent variable in step III; $X_{i}$ are the control variables; and $\varepsilon_{i}$ is the perturbation term.

## 3. Results

### 3.1. Descriptive Statistics

The general statistics and CES-D score of the grouped samples in the data set are shown in Table 2. Several preliminary findings can be drawn from Table 2:

(1) the CES-D score of informal employees (mean = 5.34, SD = 3.62) is higher than that of formal employees (mean = 4.73, SD = 3.26);
(2) the prevalence of depressive symptoms among informal employees (25.5%) is higher than that among formal employees (19.3%);
(3) the proportion of formal employees working with the Internet (66.3%) is much higher than that of informal employees (28.7%); however, Internet use shows no significant impact on the CES-D score in both the informal group (mean: 5.20 versus 5.40, SD: 3.34 versus 3.72) and formal group (mean: 4.71 versus 4.77, SD: 3.21 versus 3.36);
(4) the CES-D score of women is higher than men in both the informal group (mean: 5.65 versus 5.14, SD: 3.68 versus 3.56) and formal group (mean: 4.95 versus 4.58, SD: 3.24 versus 3.27);
(5) the relationship between age and CES-D score presents an inverted U-shaped curve, while the CES-D score of middle-aged groups is the highest, and participants in marriage have higher CES-D score in both groups than those not in marriage;
(6) the CES-D score decreases with the increase of educational years, perceived health level, personal and household annual income, perceived interpersonal relationship, and social class index.

**Figure 1:**
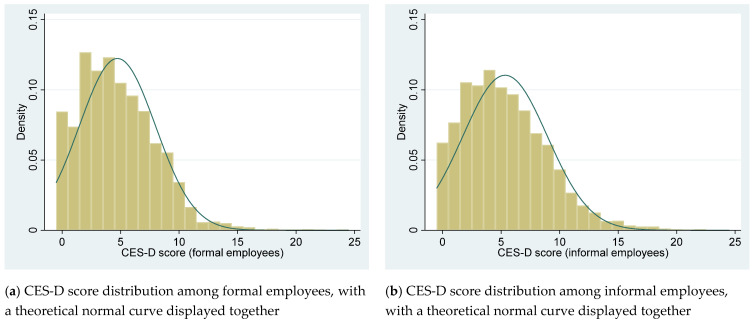
Histogram of CES-D score distribution.

**Figure 2:**
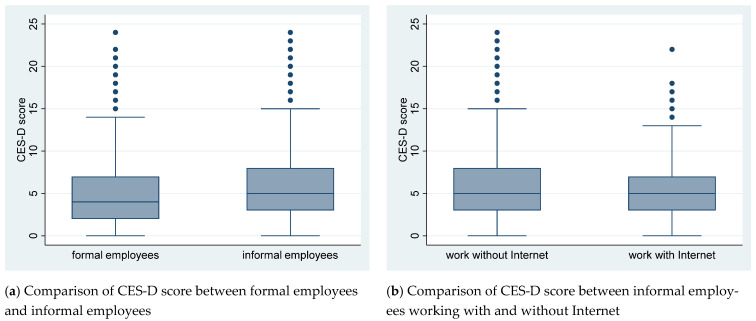
Boxplot of CES-D score comparison.

The CES-D score distribution is shown by histogram graph in Figure 1. The comparison of CES-D score between subsamples is shown by boxplot graph in Figure 2.

By visual inspection of Figure 1, both the CES-D score of formal and informal employees presents right-skewness distribution rather than normal distribution. By visual inspection of Figure 2a, the median value and range of CES-D scores among informal employees score are higher than that among formal employees. By visual inspection of Figure 2b, the CES-D score range of informal employees working without Internet is larger than that of informal employees working with Internet but the median value of the two subsamples is the same.

### 3.2. Difference between Formal and Informal Employees

Since the CES-D score of formal and informal employees all presents right-skewness distribution, it is not appropriate to use *Z* test or *t* test to compare the value between two subsamples. In this study, the nonparametric Mann–Whitney test method was used. A Mann–Whitney U test was run to determine if there was significant difference in the CES-D score between formal and informal employees. The results show that the CES-D score of formal employees (mean rank = 4181.2) and informal employees (mean rank = 4581.3) are significantly different (*z* = −6.968, *p* < 0.001).

Regression analysis was taken to validate whether the results were reliable. Both the ordered probit model and binary probit model were used and the regression results showed the two models are all significant at 1% level (*p* < 0.001). The coefficients and *p*-Value of independent variable and control variables are listed in Table 3.

As can be drawn from Table 3, informal employment is positively associated with CES-D score (*Coef* = 0.094, *p* < 0.001), which reported a higher risk of depressive symptoms among informal employees than that among formal employees. Gender is negatively associated with CES-D score (*Coef* = −0.117, *p* < 0.001), which means female employees usually have a higher risk of depressive symptoms than male employees. Age is positively associated with CES-D score (*Coef* = 0.043, *p* < 0.001) while the square of age is negatively associated with CES-D score (*Coef* = −0.062, *p* < 0.001). The relationship between age and CES-D score presents an inverted U-shaped curve, which indicates that the middle-aged people are more depressed than young and elder people. Educational years are negatively associated with CES-D score (*Coef* = −0.014, *p* < 0.001), which indicates that higher education level has an effect on decreasing the depression level of employees. Marriage has a huge negative effect on the depressive symptoms of employees (*Coef* = −0.204, *p* < 0.001). Perceived health is negatively associated with CES-D score (*Coef* = −0.247, *p* < 0.001), and the coefficient value is high, which reports a close relationship between physical and mental health. The association between CES-D score and personal annual income is not significant (*Coef* = 0.017, *p* = 0.466). Oppositely, the association between CES-D score and household annual income is negatively significant (*Coef* = −0.106, *p* < 0.001). Perceived interpersonal relationship index is negatively associated with CES-D score (*Coef* = −0.059, *p* < 0.001), and perceived social class index is negatively associated with CES-D score (*Coef* = −0.109, *p* < 0.001), which is consistent with public cognition that better interpersonal relationship and higher social class will decrease the possibility of depressive symptoms.

The regression results of the binary probit model are similar with that in ordered probit model. All variables are significant at 1% level except personal annual income (*Coef* = 0.011, *p* = 0.743), and the sign of each coefficient is consistent with that in the ordered probit model, which verifies the reliability of the regression results.

### 3.3. Impact of Internet Use among Informal Employees

A Mann–Whitney U test was run to determine if there was significant difference in CES-D score between informal employees working with and without Internet. The results show that the CES-D score of informal employees working with Internet (mean rank = 2924.9) and without Internet (mean rank = 2967.1) is not significantly different (*z* = 0.864, *p* = 0.388). As a comparative reference, the prevalence of depressive symptoms among informal employees working without Internet (26.2%) is a bit higher than that among informal employees working with Internet (23.7%), although the gap is not significant. Further, the annual income of informal employees working with Internet (mean = 50,226, SD = 31,121, Unit: CNY) is obviously higher than that of informal employees working without Internet (mean = 40,408, SD = 24,852, 1, Unit: CNY). A possible explanation is that Internet use is an external characteristic of high education, which is often related to high income, but there is no significantly direct association between Internet use and depressive symptoms among informal employees.

Regression analysis was taken to validate whether the results are reliable. Both the ordered probit model and binary probit model were used and the regression results show the two models are all significant at 1% level (*p* < 0.001). The coefficients and *p*-Value of independent variable and control variables are listed in Table 4.

As can be drawn from Table 4, Internet use is not significantly associated with the CES-D score of informal employees (*Coef* = −0.002, *p* = 0.962), which indicates that engaging in digital economy does not significantly improve the mental health status of informal employees. The association between the control variables and depressive symptoms is basically the same as in Section 3.2. The gender inequality and inverted U-shape age-depression curve still exist among informal employees. Education level, marital status, perceived health, household annual income, perceived interpersonal relationship, and social class index are negatively associated with depressive symptoms, while the personal annual income is not significantly associated with the depressive symptoms of informal employees (*Coef* = 0.027, *p* = 0.333).

The regression results of the binary probit model are similar with that in the ordered probit model. All variables are significant at 1% level except Internet use (*Coef* = −0.022, *p* = 0.640) and personal annual income (*Coef* = 0.004, *p* = 0.922), and the sign of each coefficient is consistent with that in the ordered probit model, which verifies the reliability of the regression results.

## 4. Discussion

To the best of our knowledge, this is the first study focusing on the depressive symptoms among Chinese informal employees which also concerns the impact of the digital economy. From the perspective of the difference in depressive symptoms between formal and informal employees, we draw the conclusion that informal work will obviously increase the mental health risk of employees. The results of this study are basically consistent with most previous studies in China and abroad [5,6,7,8,9,10,12,34]. A typical exception is stated in [11]. The authors do not find differences in self-perceived health (psychosocial risk factors included) status between employment profiles in Spain, except for women engaged in informal employment. The explanation may be the wide social protection coverage provided by the Spanish welfare state. The comparison with this study reveals that a labor market with a high-level social security system can greatly reduce the health inequality between informal and formal employment.

We also observed that Chinese women engaging in informal employment reported a higher risk of depressive symptoms than men, which is consistent with the finding in [34,35]. This is not only because women are naturally more sensitive and vulnerable, but also because of the gender discrimination existing in the Chinese labor market. In addition to the heavy housework, a large number of women are engaged in informal work with low income, high work hours, insufficient welfare and security, which may greatly damage their physical and mental health.

Compared with young and elder people, the middle-aged employees are more likely to suffer from depressive symptoms, which is consistent with the finding in [34]. The main reason may be the middle-aged people bear the burden of the whole family, and they need to earn enough money to raise children and support elderly parents. Furthermore, we find that people in marriage are less likely to have depression than single or divorced people, and higher education level, perceived health level, household annual income, perceived interpersonal relationship, and social class index are helpful to reduce the risk of depressive symptoms, which is the same as we expected. In particular, compared to personal income, we find that the influence of household income on depressive symptoms is more decisive. The explanation of this phenomenon might be that Chinese parents often provide a great amount of financial support for their children even after they grow up and marry. People from low-income families tend to have less financial support and more life stress, resulting in a higher possibility of depression.

From the perspective of the impact of the digital economy on informal employment, this study empirically concludes that the association between Internet use and depressive symptoms among informal employees is not significant, and the difference in prevalence of depressive symptoms between informal employees working with or without Internet is not obvious.

Previous researchers hold different views on the influence of the digital economy over labor market [15,16,17,23,24,25,26,27]. Some researchers believe that the development of informal employment in the digital economy has a positive influence on the traditional labor market. The new economic forms of informal employment enable employees to work for multiple platforms concurrently, which can improve their skills and increase their income. Besides, the new forms of informal employment create fair employment and entrepreneurship opportunities, which is conducive in promoting the employment of vulnerable groups. The elderly, women, and disabled people can find jobs more easily in the digital economy. Other researchers hold a negative opinion. They claim that the new employment forms based on digital platforms do not produce good jobs due to the irregular hours, overwork, social isolation, and lack of labor security. Platform providers tend to evade the responsibility of being employers and escape the supervision of labor laws and safety regulations, which leads to the lack of labor protection and high occupational health risk of employees and produces great unfairness between digital platform owners and workers. Our findings in this study provide more empirical support to the latter point of view that there is no significant difference in depressive symptoms between informal employees working in the digital economy and traditional sectors.

Overall, the mental health status of Chinese informal employees is still poor in the digital era. Besides the shortage in labor protection and social security coverage in traditional informal sectors, the diversified, complex, and personalized labor relations in the digital economy also make it difficult for participants to protect their labor security rights. Countermeasures should be taken to eliminate the institutional barriers and employment discrimination that affect employment inequality, and mental health-related nursing and resources should also be improved to protect the occupational and mental health of the informal employees. Nowadays, the Chinese government is striving to incorporate the new employment forms into the governance framework. The findings of this study can provide a certain reference for the designation of relevant regulations and policies.

## 5. Limitations and Future Research

The limitations of this study are stated as follows. Considering the complexity of the identification of informal employment, although the sample identification of this study has been adapted to the particular conditions in China as close as possible, there may still be misjudgment of individual cases. In the regression analysis, there may be multicollinearity between the independent variable and the control variables. Nevertheless, since the research data set contains a large number of samples, that will greatly weaken the disadvantages of individual misjudgment and model multicollinearity and will not affect the main conclusions of this study.

The future research work can be drafted from the following two aspects: (1) to extract the independent cases from CFPS panel data that change their work state in the past few years and explore the changes of their mental health status from the individual perspective; (2) to take face-to-face interview among the informal employees in representative digital industries so as to learn more about their needs and put forward more effective countermeasures to improve their mental health status.

## 6. Conclusions

This study reveals that the mental health inequality between formal and informal employees still exists in the Chinese labor market in the digital era. Compared to formal employees, informal employees are more likely to suffer from depressive symptoms, which need more public attention and psychological healthcare resources. Development of the digital economy may be conducive to increase the income of informal employees but can hardly improve their mental health status. The empirical findings in this study provide evidence for the perfection of labor market regulations and social policies in China, which indicates the necessity to consider the future working conditions of informal employment not only from the economic income and occupational safety perspective but also in terms of the mental health and well-being of employees.

## Figures and Tables

**Table 1:** Description and value range of variables in the regression model.

Type	Variable	Description
Dependent Variables	*cesd*	8-item CES-D score, between 0 and 24
	*depress*	depressive symptoms, 1: *cesd* ≥ 8; 0: *cesd* < 8
Independent Variables	*informal*	1: informal employment; 0: formal employment
	*internet*	1: work with Internet 3 days per week and above; 0: others
Control Variables	*gender*	1: male; 0: female
	*age*	values range from 18 to 65
	*age2*	*age*^2^/100, used to check if there are U-shaped features
	*edu*	years of education, 0: none; 6: primary school; 9: middle school; 12: high school; 16: college and above
	*marital*	1: married, 0: single or divorced
	*health*	perceived health, values range from 1(not healthy) to 5(very healthy)
	Income *	personal annual income
	h_income *	household annual income
	*popular*	perceived interpersonal relationship index, values range from 0 (lowest) to 10 (highest)
	*socialclass*	perceived social class, values range from 1 (lowest) to 5 (highest)

* The logarithm of *income* and *h_income* is used in the regression model.

**Table 2:** General statistics and CES-D score of the grouped samples.

	Informal Employees				Formal Employees			
Variable	N	%	Mean	S.D.	N	%	Mean	S.D.
CES-D Score	5909	100	5.34	3.62	2984	100	4.73	3.26
Depressive Symptoms (*cesd* ≥ 8)								
Yes	1506	25.5	10.21	2.49	576	19.3	9.73	2.23
No	4403	74.5	3.68	2.13	2408	80.7	3.54	2.15
Working with Internet								
Yes	1694	28.7	5.20	3.34	1979	66.3	4.71	3.21
No	4215	71.3	5.40	3.72	1005	33.7	4.77	3.36
Gender								
Male	3604	61.0	5.14	3.56	1775	59.5	4.58	3.27
Female	2305	39.0	5.65	3.68	1209	40.5	4.95	3.24
Age								
18–30 years	1785	30.2	5.28	3.42	999	33.5	4.75	3.22
31–45 years	2152	36.4	5.67	3.66	1291	43.3	4.87	3.25
>45 years	1972	33.4	5.03	3.71	694	23.3	4.44	3.32
Education								
Primary School and Below	1586	26.8	5.68	4.00	135	4.5	4.87	3.66
Middle School	2302	39.0	5.35	3.54	493	16.5	4.54	3.12
High School	1155	19.5	5.09	3.42	637	21.3	4.75	3.34
College and Above	866	14.7	5.02	3.28	1719	57.6	4.76	3.24
Marital status								
Married	4668	79.0	5.18	3.56	2319	77.7	4.65	3.18
Single/Divorced	1241	21.0	5.93	3.75	665	22.3	5.00	3.51
Perceived Health								
Q1 (very healthy)	983	16.6	4.20	3.48	391	13.1	3.47	2.76
Q2	1079	18.3	4.47	3.12	565	18.9	3.98	2.88
Q3	2715	45.9	5.51	3.49	1593	53.4	4.84	3.17
Q4	699	11.8	6.08	3.47	268	9.0	5.86	3.19
Q5 (not healthy)	433	7.3	7.85	4.31	167	5.6	7.37	4.18
Personal Annual Income								
<CNY 15,000	344	5.8	6.00	4.11	24	0.8	5.42	3.30
~CNY 30,000	1462	24.7	5.44	3.73	320	10.7	4.97	3.54
~CNY 60,000	2828	47.9	5.26	3.52	1353	45.3	4.79	3.28
~CNY 100,000	1080	18.3	5.27	3.53	861	28.9	4.71	3.23
≥CNY 100,000	195	3.3	4.95	3.48	426	14.3	4.37	3.05
Household Annual Income								
<CNY 30,000	1048	17.7	5.75	3.87	196	6.6	5.07	3.11
~CNY 60,000	2133	36.1	5.52	3.68	520	17.4	5.03	3.37
~CNY 120,000	1948	33.0	5.13	3.45	1147	38.4	4.82	3.37
~CNY 200,000	552	9.3	4.74	3.19	629	21.1	4.61	3.20
≥CNY 200,000	228	3.9	4.94	3.76	492	16.5	4.23	2.95
Interpersonal Relationship Index								
Q1 (highest)	1000	16.9	4.89	3.80	425	14.2	3.90	3.07
Q2	2670	45.2	4.93	3.32	1740	58.3	4.52	3.07
Q3	1926	32.6	5.89	3.68	737	24.7	5.52	3.45
Q4	245	4.1	6.67	3.82	72	2.4	6.46	4.29
Q5 (lowest)	68	1.2	7.72	5.16	10	0.3	6.1	4.63
Social Class Index								
Q1 (highest)	440	7.4	4.93	3.71	115	3.9	3.89	2.84
Q2	842	14.2	3.51	3.21	488	16.4	3.75	2.82
Q3	2888	48.9	5.06	3.34	1687	56.5	4.62	3.06
Q4	1066	18.0	6.09	3.75	493	16.5	5.66	3.50
Q5 (lowest)	673	11.4	6.67	4.35	201	6.7	6.19	4.32

**Table 3:** Regression results of probit model.

	Ordered Probit Model			Binary Probit Model		
Variable	Coef.	S.E.	*p*-Value	Coef.	S.E.	*p*-Value
*informal*	0.094	0.026	0.000	0.099	0.038	0.009
*gender*	−0.117	0.024	0.000	−0.109	0.033	0.001
*age*	0.043	0.008	0.000	0.051	0.011	0.000
*age2*	−0.062	0.009	0.000	−0.068	0.013	0.000
*edu*	−0.014	0.003	0.000	−0.014	0.004	0.002
*marital*	−0.204	0.032	0.000	−0.270	0.044	0.000
*health*	−0.247	0.011	0.000	−0.250	0.015	0.000
*income*	0.017	0.023	0.466	0.011	0.033	0.743
*h_income*	−0.106	0.018	0.000	−0.158	0.025	0.000
*popular*	−0.059	0.006	0.000	−0.059	0.009	0.000
*socialclass*	−0.109	0.011	0.000	−0.137	0.016	0.000

**Table 4:** Regression results of probit model.

	Ordered Probit Model			Binary Probit Model		
Variable	Coef.	S.E.	*p*-Value	Coef.	S.E.	*p*-Value
*internet*	−0.002	0.034	0.962	−0.022	0.047	0.640
*gender*	−0.129	0.030	0.000	−0.112	0.041	0.006
*age*	0.044	0.009	0.000	0.051	0.012	0.000
*age2*	−0.061	0.011	0.000	−0.069	0.015	0.000
*edu*	−0.018	0.004	0.000	−0.017	0.005	0.001
*marital*	−0.264	0.039	0.000	−0.279	0.053	0.000
*health*	−0.234	0.013	0.000	−0.231	0.018	0.000
*income*	0.027	0.028	0.333	0.004	0.040	0.922
*h_income*	−0.115	0.021	0.000	−0.153	0.030	0.000
*popular*	−0.049	0.007	0.000	−0.048	0.010	0.000
*socialclass*	−0.095	0.013	0.000	−0.120	0.018	0.000

## Data Availability

Original data in this study are obtained from the Institute of Social Science Survey of Peking University and are available at http://www.isss.pku.edu.cn/cfps/index.htm (registration and approval needed, accessed on 11 May 2021).
